# Evaluation of the effects of diosmin in cyclophosphamide-induced nephrotoxicity: an experimental animal study

**DOI:** 10.1007/s00210-026-05349-y

**Published:** 2026-05-01

**Authors:** Betul Yalcin, Kubra Tugce Kalkan, Eda Koseoglu, Fazile Canturk Tan, Sedat Carkit, Gozde Ozge Onder, Arzu Yay

**Affiliations:** 1 Department of Histology and Embryology, Faculty of Medicine, Adıyaman University, 02040 Adıyaman, Turkey; 2 Department of Histology and Embryology, Faculty of Medicine, Kırşehir Ahi Evran University, 40100 Kırşehir, Turkey; 3 Department of Histology and Embryology, Faculty of Medicine, Erciyes University, 38039 Kayseri, Turkey; 4 Department of Biophysics, Faculty of Medicine, Erciyes University, 38039 Kayseri, Turkey; 5 Department of General Surgery, Faculty of Medicine, Erciyes University, 38039 Kayseri, Turkey; 6 Genome and Stem Cell Center (GENKOK), Erciyes University, Kayseri, Turkey

**Keywords:** Cyclophosphamide, Diosmin, Inflammation, Apoptosis, DNA damage

## Abstract

Cyclophosphamide (CYC) is a well-established nephrotoxic agent widely used in the treatment of cancer and various immunosuppressive disorders. Diosmin, a bioactive flavonoid, has been reported to reduce drug-induced adverse effects. This study evaluates the effects of diosmin on CYC-induced nephrotoxicity and explores potential mechanisms involved in CYC-related renal damage. Thirty-two male rats were randomly assigned to four experimental groups: control, diosmin, CYC, and CYC + diosmin. Diosmin (100 mg/kg) was administered once daily for 15 consecutive days, while cyclophosphamide (200 mg/kg) was given as a single dose on the 8th day of the experimental period. Diosmin was associated with attenuation of CYC-induced pathological alterations in renal tissue architecture. It reduced the immunoreactivity of pro-inflammatory mediators (IL-1β, IL-6, TNF-α, and iNOS) and modulated apoptosis by decreasing the pro-apoptotic marker BAX and increasing the anti-apoptotic marker Bcl-2. Although CYC treatment suppressed mTOR and increased SIRT1 immunoreactivity, diosmin co-administration attenuated these alterations. Additionally, markers of renal DNA damage were reduced. Overall, the findings suggest that diosmin may exert favorable effects in CYC-induced nephrotoxicity in this experimental model.

**Graphical Abstract:**

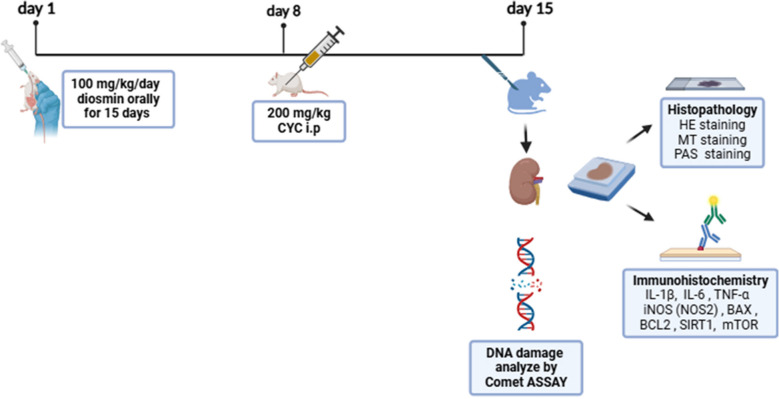

## Introduction

Cyclophosphamide (CYC), a well-established alkylating agent, has long been utilized in the management of various neoplastic and non-neoplastic conditions due to its potent immunosuppressive and cytotoxic properties (Jiang et al. 2019; Ahlmann and Hempel 2016). Despite its proven therapeutic efficacy across a broad spectrum of diseases, the clinical application of cyclophosphamide is often restricted due to its adverse effect profile, with nephrotoxicity being one of the most significant concerns (Zhai et al. 2018). In hepatocytes, CYC undergoes bioactivation via cytochrome P450 enzymes to yield two major reactive metabolites: phosphoramide mustard and acrolein (Jiang et al. 2020). Acrolein, known to trigger the synthesis of reactive oxygen species (ROS) (Moghe et al. 2015), impairs cellular defense mechanisms and subsequently leads to various physiological and morphological alterations (Çağlayan et al. 2018; Kim et al. 2014). The generation of ROS acts as a key initiating event that triggers a cascade of cellular disturbances, including lipid peroxidation of cell membranes, interaction with critical cellular macromolecules, disruption of normal cellular functions, and activation of various intracellular signaling pathways (Mahmoud 2014; Nafees et al. 2015). In response to acrolein exposure, the resulting ROS can modulate inflammatory and apoptotic signaling pathways, thereby exacerbating oxidative stress and enhancing the risk of renal tissue injury (ALHaithloul et al. 2019; Lin et al. 2017). ROS induce prolonged inflammation via NF-κB activation (Zeng et al. 2022), which, in turn, stimulates pro-inflammatory cytokines including interleukin-1β (IL-1β) and tumor necrosis factor α (TNF-α). These cytokines are involved in controlling both the inflammatory response and apoptosis (Iqubal et al. 2019; Zeng et al. 2022). Activation of NF-κB and the subsequent release of TNF-α and IL-1β lead to transcriptional upregulation of inducible nitric oxide synthase (iNOS), resulting in increased nitric oxide and COX-2 mediated prostaglandin production and further amplification of the inflammatory response (Mahmoud et al., 2014; Nafees et al. 2015). Overproduction of nitric oxide further exacerbates oxidative stress by depleting intracellular glutathione levels, thereby increasing cellular susceptibility to ROS-mediated damage (Kandemir et al. 2019).

To mitigate CYC-associated adverse effects, several strategies have been proposed, including antioxidant therapy, alternative CYC analogs, and combination regimens involving low-dose CYC and other anticancer agents. However, these approaches remain under active investigation as mechanistic understanding of CYC toxicity continues to evolve (Casak et al. 2011; Abdallah et al. 2026; Alruhaimi et al. 2026). Despite ﻿substantial experimental evidence demonstrating CYC-induced hepatorenal toxicity and the exploration of emerging therapeutic approaches, such as mesenchymal stem cell-derived exosomes (Abdallah et al. 2026), clinically effective and broadly applicable protective strategies remain limited. This gap underscores the need for the development of novel preventive and therapeutic interventions (Basu et al. 2014; Patwa et al. 2020).

Flavonoids, a class of phytochemicals produced by various plants, serve a protective function, preventing infection, injury, and stress (Ali et al. 2021a, b; Panche et al. 2016). Diosmin, a flavone glycoside originating from hesperidin, a flavonoid abundant in citrus fruits, represents a significant pharmacological agent (Szymański et al. 2016). It has a number of pharmacological benefits, including anti-inflammatory, anticancer, antioxidant, anti-diabetic, and antifibrotic characteristics (Gerges et al. 2022; Mirzaee et al. 2019). Diosmin (Daflon-500®) is presented in film-coated tablets comprising 450 mg diosmin and 50 mg hesperidin, a flavonoid fraction derived from the plant Rutaceae aurantiae (Ramelet et al. 2001). The combined use of these compounds has been demonstrated to potentiate their effects (Olatunji et al. 2022). In experimental models of acrylamide-induced organ toxicity, the combination demonstrated superior protective efficacy by mitigating oxidative stress, lipid peroxidation, and genotoxic damage. Clinically, it has been widely used for the treatment of vascular disorders such as varicose veins, chronic venous insufficiency, and hemorrhoids, owing to its venotonic and anti-inflammatory properties (Srinivasan et al. 2019). Following oral administration, diosmin undergoes conversion to diosmetin, subsequently being absorbed and eliminated from the body via the urinary system (Chen et al. 2019; Gerges et al. 2022). Several studies have suggested that diosmin may confer protective effects against nephrotoxicity induced by various pharmaceutical agents (Elhelaly et al. 2019; Anwer et al. 2023; Nadeem et al. 2023). Due to its anti-inflammatory properties, diosmin has been shown to significantly reduce levels of pro-inflammatory cytokines in models of hepatic, renal, and cardiac injury induced by methotrexate (Abdel-Daim et al. 2017). Furthermore, in a model of doxorubicin-induced renal toxicity, diosmin administration led to improved renal biochemical parameters and ameliorated histopathological damage (Ali et al. 2021b).

This study was designed to evaluate the histological and immunohistochemical alterations associated with CYC-induced renal toxicity and to investigate the protective effects of diosmin, focusing on inflammatory and apoptotic pathways and DNA damage assessed by the comet assay.

## Materials and methods

### Chemicals

Daflon (500 mg/tablet, Les Laboratoires Servier, France) was provided by a qualified pharmaceutical company. A solution was prepared by dissolving the drug in 5 mL of deionized water thereby creating a solution with a 100 mg/mL concentration (Lamidi et al. 2021; Olatunji et al. 2022).

### Animal care

The Erciyes University Local Ethics Committee approved the experimental protocol (Protocol No: 2023–23/082). The care and use of the animals were carried out in accordance with the ARRIVE guidelines for animal research ethics, the UK Animals (Scientific Procedures) Act 1986, the European Directive 2010/63/EU, and the National Institutes of Health Guide for the Care and Use of Laboratory Animals. For the experimental study, 32 adult male Wistar albino rats were obtained from Erciyes University Experimental Research Application and Research Center (DEKAM, Kayseri, Türkiye). Throughout the course of the experiment, the animals were accommodated in disinfected cages at standard conditions (22–24 °C, 12-h light and 12-h dark period). Additionally, the rats were permitted ad libitum accessibility to water and pellets.

### Experimental design

In the study, four randomly selected groups of eight male rats (age 10–12 weeks, weight 200–250 g**)** each were included and they were treated in accordance with the following protocol:

- Group 1 (control) (n = 8): 0.9% saline was administered to the rats orally for 15 days and intraperitoneally on day 8.
- Group 2 (diosmin) (n = 8): 100 mg/kg diosmin was administered to the rats orally for 15 days.
- Group 3 (CYC) (*n* = 8): on day 8, 200 mg/kg CYC was administered intraperitoneally to the rats (Tohamy et al. 2021).
- Group 4 (CYC + diosmin) (*n* = 8): 100 mg/kg diosmin was administered to the rats orally for 15 days, and on day 8, 200 mg/kg CYC was administered intraperitoneally to the rats (Germoush 2016; Abogresha et al. 2021).

The quantity and duration of diosmin administered to each animal were determined based on previously published research findings (Germoush 2016; Abogresha et al. 2021). Cyclophosphamide (CYC) (Endoxan 1 g/ampoule) was acquired from Baxter Oncology GmbH (Halle, Germany). The quantity and the administration route of CYC were selected based on previous studies (Tohamy et al. 2021; Adeyemi et al. 2024).

### Tissue sample collection

On the 16th day, general anesthesia was induced with combination of ketamine and xylazine (60 mg/kg and 10 mg/kg, intraperitoneally), and the rats were subsequently euthanized via cervical dislocation. For histological and immunohistochemical applications, the left kidney tissues were preserved in a 10% buffered formaldehyde solution, and for the comet study, the right kidney tissues were retained at − 80 °C. No mortality was observed in any experimental group.

### Histopathological examination

For histological analysis, formaldehyde-fixed kidney tissue samples were processed for histological evaluation. Initially, increasing concentrations of ethanol solutions were employed for 1 h each to gradually eliminate water content from the tissues. Subsequently, they were subjected to xylene three times in order to achieve the desired transparency. The tissues were immersed in liquid paraffin for a minimum of 3 h to facilitate the penetration of the paraffin into the tissue at 60 °C, after which a paraffin block was performed. In order to ascertain whether any morphological alterations had occurred, hematoxylin–eosin (H&E), Masson’s trichrome (MT), and Periodic acid–Schiff (PAS) were applied to the gently prepared sections.

### H&E staining

Following deparaffinization at 60 °C for at least 2 h, rehydration was performed with decreasing ethanol concentrations (100%, 95%, 90%, 80%, 70%), and the sections were washed with tap water. H&E-stained sections were subjected to an increasing ethanol series and xylene. The entellan-mounted sections were subjected to microscopic examination, and images were captured (Olympus BX51, Tokyo, Japan). This staining provides information on overall renal tissue architecture and cellular morphology (Tong et al. 2024).

### MT staining

Following deparaffinization and rehydration of the sections, the nuclei were stained with hematoxylin. The sections were immersed in acid fuchsin for 2 min and subsequently washed. They were incubated in phosphomolybdic acid for 5 min, then dried for an appropriate period. Following this, they were incubated in an aniline blue solution for 5 min. Following washing of the sections, they were subjected to acetic acid, an increasing series of alcohols, xylene, mounted, and examined under a light microscope. This staining provides information on collagen deposition and interstitial fibrosis (Tong et al. 2024).

### PAS staining

Following deparaffinization and rehydration, the sections were washed with tap water and treated with freshly prepared periodic acid for 5 min and subsequently washed with distilled water. The sections were then incubated in Schiff’s reagent in the absence of light for 30 min and washed under running tap water. Subsequently, the sections were counterstained with hematoxylin. The stained sections were dehydrated, cleared in xylene, mounted with Entellan®, and examined under a light microscope. This staining provides information on basement membrane integrity and polysaccharide-rich structures in renal tissue (Tong et al. 2024).

For histopathological evaluation, renal tissues from eight animals in each group were examined. Three renal sections per animal were evaluated, and ten non-overlapping microscopic fields per section were assessed at × 40 magnification (Yalcin et al. 2024). To minimize potential bias, histological evaluations were conducted in a blinded manner by independent researchers unaware of the experimental group allocations.

### Immunohistochemistry

To qualitatively assess the immunoreactivity intensity of the primary antibodies, the avidin–biotin-peroxidase method was employed in rat kidney tissues. The sections from paraffin blocks were subjected to a series of treatments, deparaffinized in xylene, rehydrated through descending ethanol concentrations, and rinsed in distilled water. Endogenous peroxidase activity was blocked with 3% hydrogen peroxide to minimize background staining of the sections, followed by three washes with PBS. Subsequently, for 10 min, sodium citrate buffer was treated to the sections in the microwave at 95 °C, thereby facilitating antigen retrieval. They were allowed to cool in the same buffer solution for 10 min. Following PBS washing, Ultra V Block was applied for 10 min to cover areas outside the antigenic regions. Over the course of the night at 4 °C, the primary antibodies against IL-1β (1:200, BT-AP04469, Bioassay Technology Laboratory, Zhejiang, China), IL-6 (1:300, bs-0782R, Bioss, Woburn, USA), TNF-α (1:500, NB600-587, Novus Biologicals, Centennial, USA), iNOS (NOS2) (1:200, BT-AP06109), BAX (1:200, sc-23959, Santa Cruz, Oregon, USA), Bcl-2 (1:50, PA511379, Invitrogen), SIRT1 (1:500, NBP1-51,641, Novus Biologicals, Centennial, USA), and mTOR (1:100, 7C10, Cell Signaling, Massachusetts, USA) were applied to the sections. On the following day, following the PBS washing procedure, the sections were treated with biotinylated secondary antibodies and streptavidin-conjugated peroxidase in that order, and finally with chromogen (3,3′-diaminobenzidine tetrahydrochloride (DAB)). After Gill hematoxylin counterstaining, the sections were subjected to washing with distilled water, dehydrated through ascending ethanol concentrations, xylene, and entellan coating, and finally were examined under a light microscope (Olympus BX51).

Immunoreactivity intensity was quantified by analyzing ten randomly selected, non-overlapping cortical regions per animal using ImageJ software (ImageJ, Version 1.8.0, Bethesda, MD) (Baran et al. 2022; Yalcin et al. 2024).

### Detection of DNA damage by comet assay

Approximately 2 g of kidney tissue from each rat was placed in a plate and suspended in 3 mL of cold calcium- and magnesium-free PBS. Cell suspensions were obtained by finely mincing and homogenizing the tissue samples, which were then transferred to test tubes. Meanwhile, a solution including 1000 μL agarose (0.8% low melting point) and 100 μL supernatant was applied to slides pre-coated with agarose (0.5% normal melting point) and mounted with coverslips. The agarose on the slides was frozen in an icebox at 4 °C, and the coverslips were gently removed. The recently made cold lysis buffer (TBE, 25 g SDS) was applied to the slides maintained at 4 °C for 4 min. Subsequently, the slides were placed in a horizontal electrophoresis tank containing fresh electrophoresis buffer for 10 min, facilitating DNA relaxation. Electrophoresis was performed on these slides for 2 min at 64 V and 250 mA, after which they were rinsed with distilled water to eliminate residual ions and detergents. Subsequently, the remaining stages of the analysis were conducted under dark conditions. The ethidium bromide-stained sections, covered with coverslips, were prepared for observation via fluorescence microscopy. A total of 50 cells were randomly selected for analysis using the CASP software program (CASP-1.2.2; Windows 2010), with images captured at × 20 magnification in ten different fields from each rat. In this method, cells with damaged DNA that exhibited a “comet” pattern migrating out of the cell were identified as damaged, whereas cells that lacked this pattern were considered undamaged (Baran et al. 2022; Yalcin et al. 2023).

To minimize potential bias, comet assay analyses were conducted in a blinded manner by independent researchers unaware of the experimental group allocations.

### Statistical analysis

A statistical analysis of the data set was executed utilizing the GraphPad Prism software (GraphPad Inc., version 9.0). The data were subjected to an assessment of their normal distribution via the utilization of the Shapiro–Wilk and Kolmogorov–Smirnov tests. In cases where the data conformed to a normal distribution, one-way analysis of variance (ANOVA) followed by Tukey’s post hoc test was employed for the comparison of quantitative variables. In cases where the data displayed an abnormal distribution, the Kruskal–Wallis test followed by Dunn’s post hoc test was employed for the comparison of quantitative variables. Depending on the data distribution, values are presented as mean ± standard deviation (SD) or median (Q1–Q3). The analysis of statistical data is defined as statistically significant when the *p*-value is less than 0.05.

## Results

### Renal histopathological alterations

**Fig. 1:**
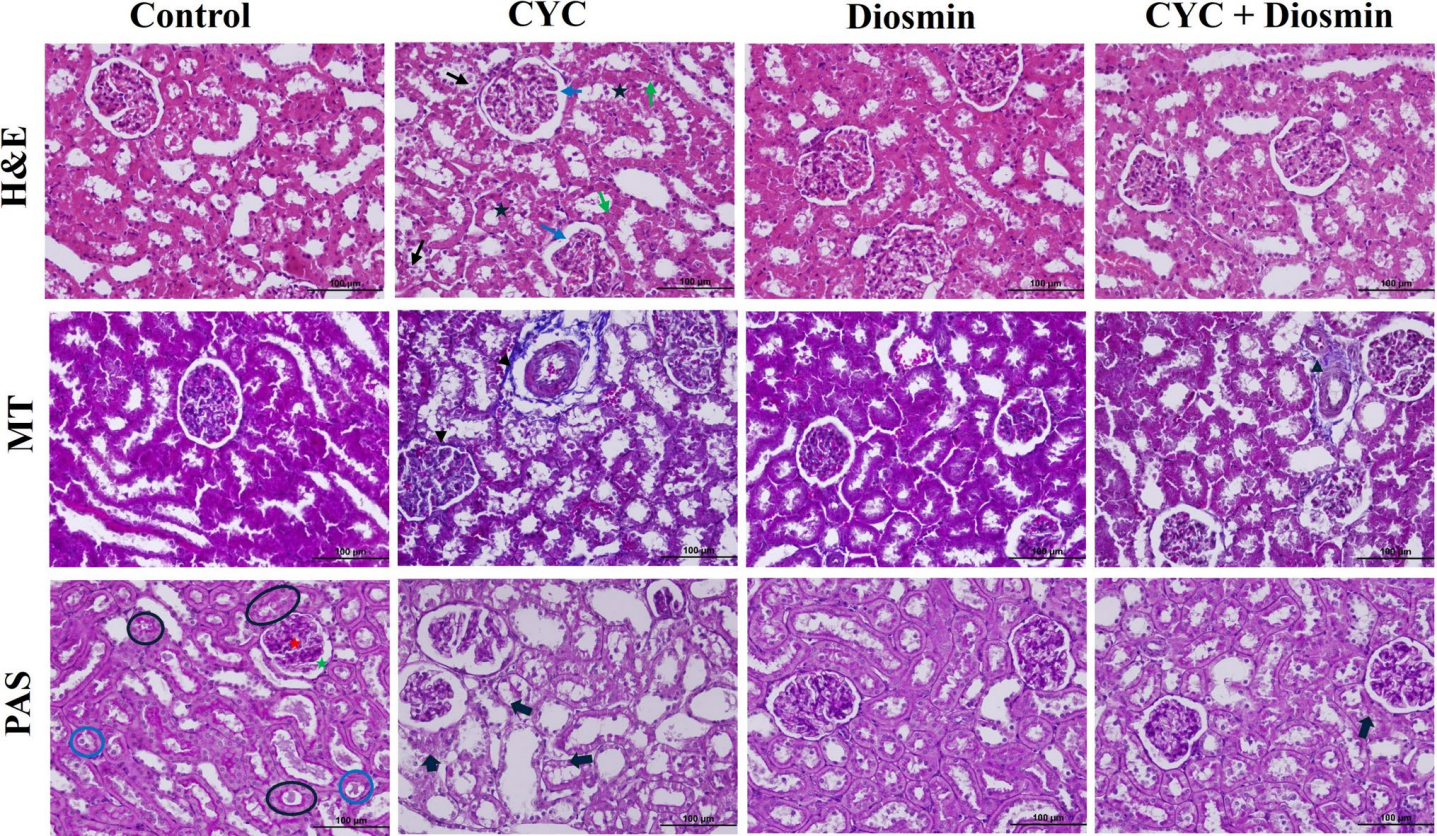
Hematoxilen and eosin (H&E), Masson’s trichrome (MT), and Periodic acid–Schiff (PAS) stained kidney sections of control, CYC, diosmin, and CYC + diosmin groups. Sections showing proximal tubules (black circle), distal tubules (blue circle), Bowman’s capsule (green star), glomeruli (red star), vacuolization (black arrow), necrosis of tubular epithelial cells (green arrow), desquamation of epithelial cells (star), enlargement of Bowman’s capsule (blue arrow), fibrosis in MT-stained sections (arrowhead), and loss of brush border in PAS-stained sections (thick arrow) (H&E, MT and PAS staining, original magnification =  × 40; scale bar = 100 μm, Olympus, Japan). H&E-stained renal sections showing representative histopathological alterations in the CYC-treated group, including enlargement of Bowman’s capsule, tubular epithelial cell vacuolization and necrosis, luminal dilatation, and epithelial cell desquamation. The CYC + diosmin-treated group exhibited preservation of renal tubular and glomerular morphology. MT-stained renal sections from the CYC-treated group showing increased connective tissue deposition surrounding the glomeruli and glomerular capillaries. The CYC + diosmin-treated group exhibited a staining pattern comparable to that of the control and diosmin groups. PAS-stained renal sections from the CYC-treated group showing reduced PAS positivity in the tubular basement membranes and disruption of brush border structures. The CYC + diosmin-treated group showed comparatively preserved PAS staining patterns

The influence of diosmin on CYC-induced nephrotoxicity was assessed using H&E, MT, and PAS staining (Fig. 1). The H&E-stained control group rats’ kidney sections exhibited normal histomorphology, displaying well-preserved proximal and distal tubules, glomeruli, and interstitial tissue. The CYC group rats’ kidney sections displayed the following pathological alterations: enlargement of Bowman’s capsule, vacuolization and necrosis of tubular epithelial cells, luminal dilatation in tubules, and desquamation of epithelial cells. These alterations were not observed in the diosmin-treated group rats’ kidney sections. The CYC + diosmin group rats’ kidney sections showed marked attenuation of the detrimental effects evident in the tubules of the CYC group (Fig. 1).

MT staining is a valuable tool for assessing the quantity and distribution of collagen in formalin-fixed tissue sections. The control group rats’ MT-stained kidney sections exhibited no alterations in the quantity of collagen fibers and were consistent with normal renal histology. However, the blue-stained connective tissue areas observed in the CYC group rats’ kidney sections were noted to be particularly concentrated around both the glomeruli and the glomerular capillaries. The quantity and distribution patterns of collagen fibers in the CYC + diosmin group demonstrated a similarity to those detected in the control and diosmin-only groups (Fig. 1).

As evidenced by PAS-stained sections, PAS reactivity was clearly observed in the basement membranes of renal tubules and the brush border in the control group. There was a reduction in PAS positivity in the tubular basement membranes, accompanied by disruption of the brush border in the CYC group. The detected pathological alterations in PAS-stained kidney sections were markedly attenuated in the CYC + diosmin group, with restoration of PAS reactivity (Fig. 1).

### Immunohistochemical findings

**Fig. 2:**
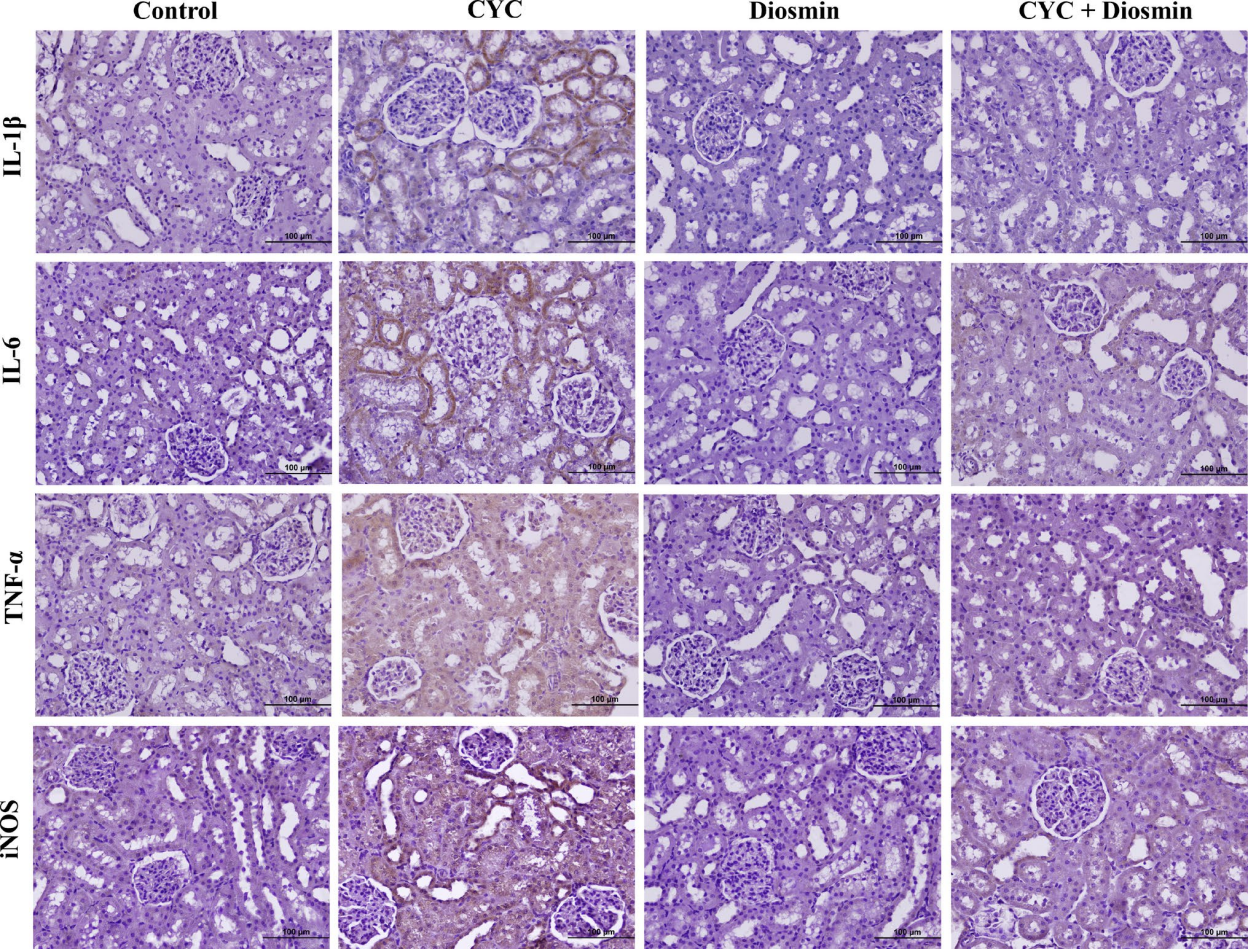
Immunoreactivity of inflammatory markers (IL-1β, IL-6, TNF-α, and iNOS) in rats’ kidney tissue of all experimental groups: control group, CYC group, diosmin group, and CYC + diosmin group. The presence of brown staining is considered an indicator of positivity (original magnification =  × 40; scale bar = 100 μm, Olympus, Japan)

**Fig. 3:**
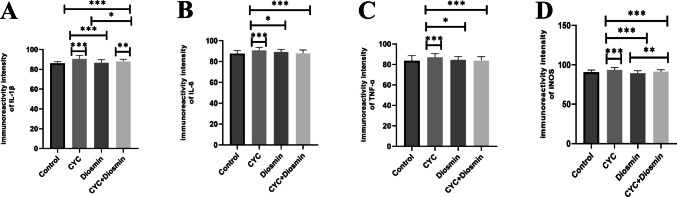
The bar graphs present a summary of the observed immunoreactivity intensity of IL-1β (**A**), IL-6 (**B**), TNF-α (**C**), and iNOS (**D**) in rats’ kidney tissue of all experimental groups: control group, CYC group, diosmin group, and CYC + diosmin group.  *n *= 8 per group. Renal IL-1β, TNF-α, and iNOS values are presented as means ± SD. IL-6 values are presented as median (Q1–Q3). Asterisks above the bars indicate statistically significant differences compared with the control group, whereas two-ended brackets denote statistically significant pairwise comparisons between the connected experimental groups. The presence of statistical significance is denoted by the use of asterisks: *p < 0.05, **p < 0.01, ***p < 0.001

Figure 2 demonstrates the contribution of diosmin to the pro-inflammatory markers’ activity. Immunoreactivity intensities of IL-1β, IL-6, TNF-α, and iNOS were markedly increased in the kidney sections from the CYC-treated group compared with the control group. In contrast, IL-1β, IL-6, TNF-α, and iNOS immunoreactivity intensities were significantly reduced in the CYC + diosmin group compared with the CYC group (*p* < 0.001). Furthermore, significant differences were observed when the CYC group was compared with all other groups (Figs. 2 and 3).

**Fig. 4:**
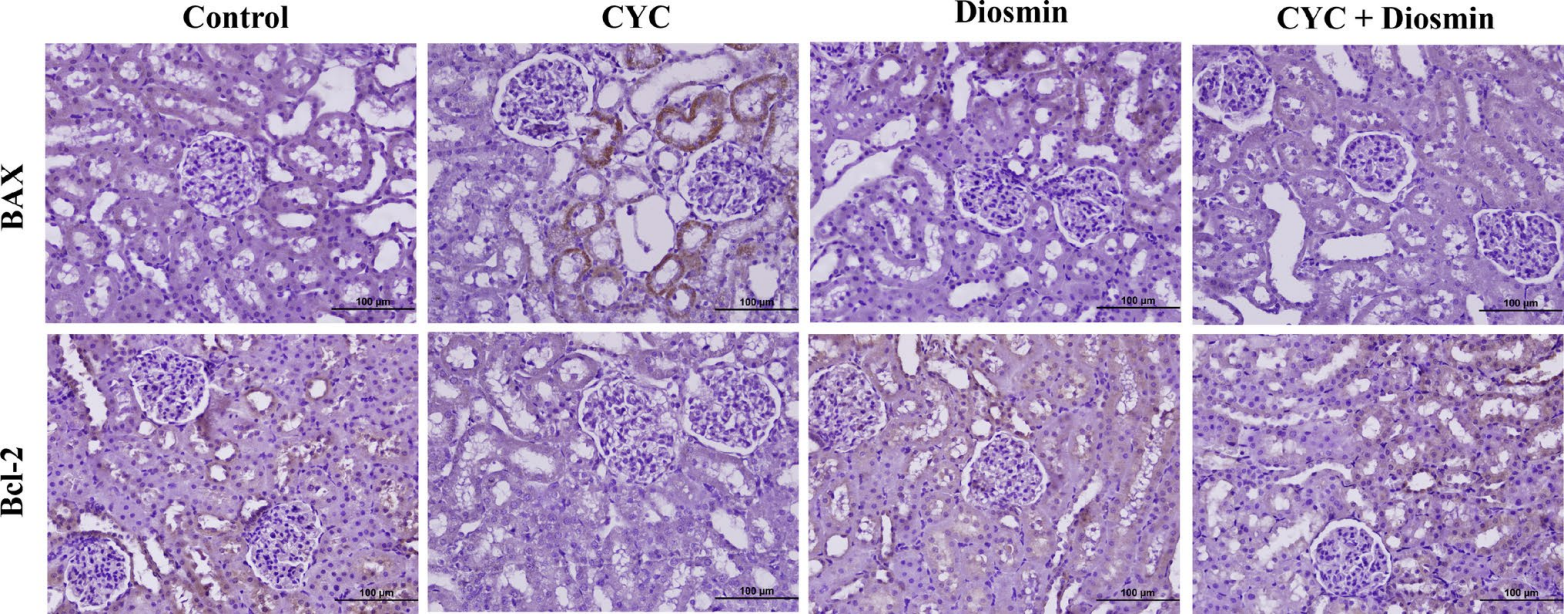
Immunoreactivity of apoptotic markers (BAX and Bcl-2) in rats’ kidney tissue of all experimental groups: control group, CYC group, diosmin group, and CYC + diosmin group. The presence of brown staining is considered an indicator of positivity (original magnification =  × 40; scale bar = 100 μm, Olympus, Japan)

**Fig. 5:**
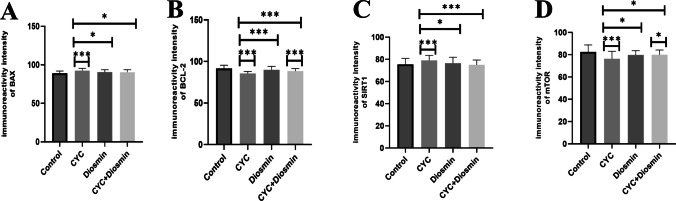
The bar graphs present a summary of the observed immunoreactivity intensity of BAX (**A**), Bcl-2 (**B**), SIRT1 (**C**), and mTOR (**D**) in rats’ kidney tissue of all experimental groups: control group, CYC group, diosmin group, and CYC + diosmin group. *n* = 8 per group. Renal SIRT1 and mTOR values are presented as median (Q1–Q3). Asterisks above the bars indicate statistically significant differences compared with the control group, whereas two-ended brackets denote statistically significant pairwise comparisons between the connected experimental groups. The presence of statistical significance is denoted by the use of asterisks: *p < 0.05, **p < 0.01, ***p < 0.001

The progression of CYC-induced nephrotoxicity is associated with apoptosis. The effect of diosmin on BAX and Bcl-2 immunoreactivity intensity was examined in CYC-treated rats. In renal tubular epithelial cells, the intensity of cytoplasmic BAX and Bcl-2 immunoreactivity was weak in the control group. The CYC group had a substantial elevation in BAX, accompanied by a marked reduction in Bcl-2 immunoreactivity intensity. In comparison with the CYC group, diosmin treatment reduced BAX immunoreactivity (*p* < 0.05) while increasing Bcl-2 immunoreactivity intensity (*p* < 0.001) (Figs. 4 and 5).

**Fig. 6:**
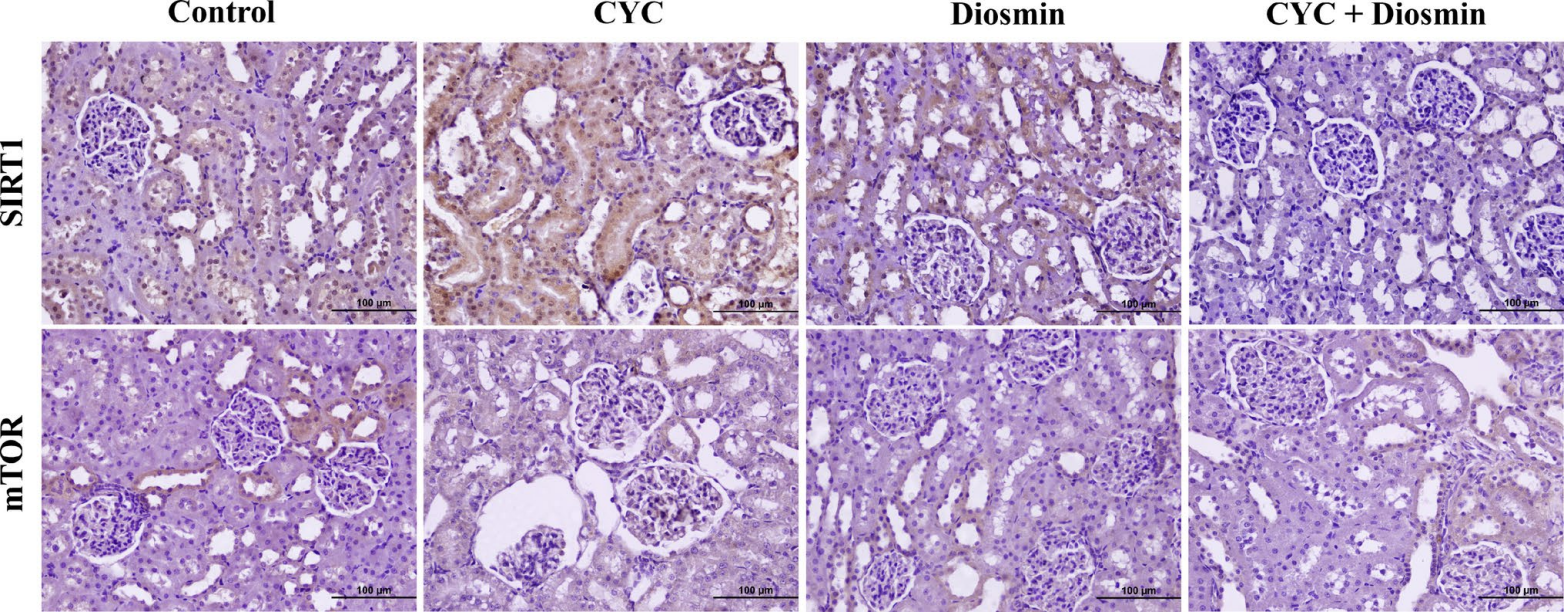
Immunoreactivity of mTOR and SIRT1 proteins in rats’ kidney tissue of all experimental groups: control group, CYC group, diosmin group, and CYC + diosmin group. The presence of brown staining is considered an indicator of positivity (original magnification = ×40; scale bar = 100 μm, Olympus, Japan)

In order to ascertain the localization and immunoreactivity of mTOR and SIRT1 proteins in the diosmin-treated kidney, the immunohistochemical technique was employed. mTOR immunoreactivity was observed in the renal tubular epithelium, with the lowest intensity detected in the CYC group and the highest intensity in the control group. The CYC + diosmin group showed significantly increased mTOR immunoreactivity intensity compared with the CYC group (*p* < 0.05) (Fig. 5). SIRT1 protein was observed in both the nuclei and cytoplasm of renal cells. In the CYC group, the intensity of SIRT1 immunoreactivity was significantly elevated compared with the control and CYC + diosmin groups (*p* < 0.001). No statistically significant differences were observed among the remaining groups (*p* > 0.05) (Fig. 6).

### Assessment of comet assay results

This study was designed to assess the contribution of diosmin to CYC-induced renal DNA damage at the cellular level using the neutral comet assay. Comet assay for each results is presented in Table 1. In the CYC group, comet parameters were increased except for Head DNA. Head DNA values were significantly reduced in both the CYC and CYC + diosmin groups compared with the control group (*p* < 0.001). The reduction in Head DNA was more pronounced in the CYC group than in the CYC + diosmin group (*p* < 0.01).

**Table 1:** The effect of CYC and diosmin on renal comet assay parameters in all experimental groups: control group, CYC group, diosmin group, and CYC + diosmin group

Commet assay parameters	Control median (Q1-Q3)	CYC Median (Q1-Q3)	Diosmin median (Q1-Q3)	CYC+Diosmin median (Q1-Q3)	p
Groups					
Head DNA (%)	96,00 (95,00–97,00)^a^	68,50 (65,75-72,00)^b^	88,50 (82,00–96,00)^a^	76,00 (72,50–79,25)^c^	<0,0001
Tail DNA (%)	4,00 (3,00–5,00)^a^	31,50 (28,00–34,25)^b^	11,50 (4,75-17,00)^a^	24,00 (20,75-17,00)^c^	<0,0001
L Head (μm)	127,0 (119,0–141,5)^a^	156,5 (89,00–175,0)^b^	122,0 (91,00–139,0)^ac^	146,0 (81,00–165,0)^c^	<0,0001
L Tail (μm)	30,50 (23,75-35,00)^a^	145,5 (117,5–211,5)^b^	47,00 (34,00–62,25)^a^	129,0 (254,8–329,0)^b^	<0,0001
L Comet (μm)	160,5 (142,0-174,3)^a^	308,5 (254,3–379,3)^b^	184,0 (158,0–221,8)^a^	295,0 (254,8–329,0)^b^	<0,0001
TM	1,00 (1,00-2,00)^a^	44,50 (31,00–62,25)^b^	12,00 (5,00–21,25)^c^	31,00 (19,00–37,75)^d^	<0,0001
OTM	2,00 (2,00–3.00^a^	34,50 (25,75–44.25)^b^	19,00 (15,00–23.25)^c^	23.50 (15,75–31.00)^c^	<0,0001

In each line, the identical letters signified the similarity between the groups, and the disparate letters indicated the dissimilarity between them. Renal comet assay parameters are presented as median (Q1–Q3). *n* = 8 per group. Head DNA, % DNA in the head; Tail DNA, % DNA in the tail; L Head, length head; L Tail, length tail; L Comet, length comet; TM, tail moment; OTM, olive tail moment

**Fig. 7:**
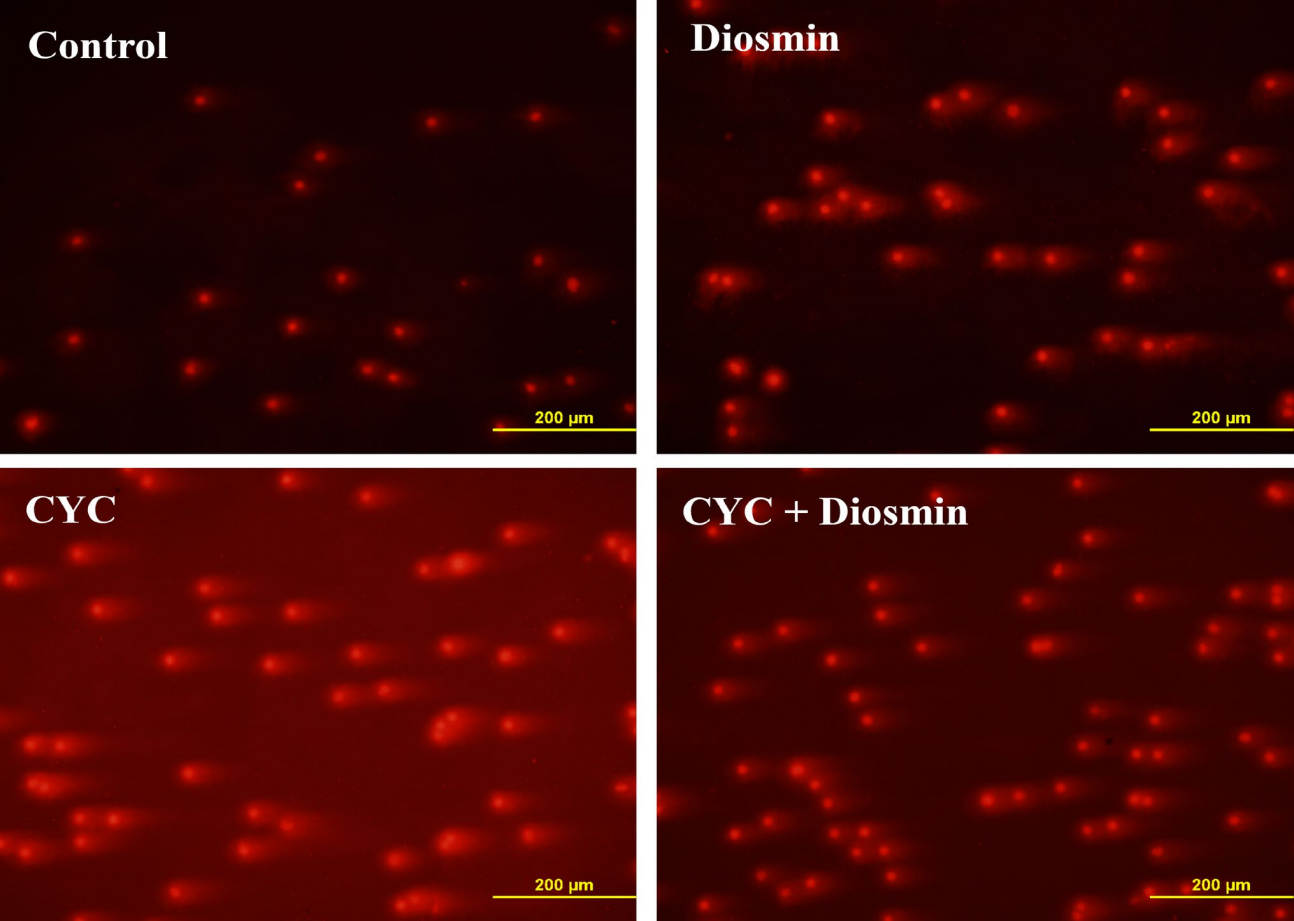
Photographs of all experimental groups visualized with a fluorescent microscope from ethidium bromide-stained sections of cellular DNA damage in kidney tissue. Control Tail DNA 4%, CYC Tail DNA 31.50%, diosmin Tail DNA 11.50%, and CYC + diosmin Tail DNA 24% (original magnification =  × 20; scale bar = 200 μm, Olympus, Japan)

Tail DNA values were significantly increased in the CYC and CYC + diosmin groups compared with the control group. Compared with the CYC group, the CYC + diosmin group showed significant differences, characterized by increased Head DNA values and decreased Tail DNA values (*p* < 0.01). L-head, L-tail, and L-comet values were higher in the CYC group than in the control and diosmin groups. All three parameters were reduced in the CYC + diosmin group compared with the CYC group. Nevertheless, only the L-head values reached statistical significance (*p* < 0.001). The TM values showed a statistically significant decrease in the CYC + diosmin group compared with the CYC group (*p* < 0.05). The OTM values were significantly increased in the CYC group compared with both the CYC + diosmin (*p* < 0.01) and diosmin (*p* < 0.001) groups. Additionally, the CYC + diosmin and diosmin groups showed similar OTM values. Overall, analysis of comet parameters indicates that diosmin treatment attenuated CYC-induced DNA damage (Fig. 7).

## Discussion

In recent years, diosmin has been identified as exhibiting significant beneficial effects in various models of tissue injury; nevertheless, the precise mechanisms underlying these effects remain incompletely understood. To the best of our knowledge, limited data are available regarding the effects of diosmin against CYC-induced renal injury, with a particular focus on the molecular mechanisms involved in its pathogenesis. CYC, frequently used as an alkylating anticancer agent and immunosuppressant, is associated with nephrotoxicity, one of its most concerning adverse effects, representing a major clinical concern for patients undergoing treatment (Ahlmann and Hempel 2016; Chiruvella et al. 2020).

CYC-induced nephrotoxicity can be attributed to multiple mechanisms, including oxidative stress, inflammation, apoptosis, and oxidative DNA damage (Çağlayan et al. 2018). In this study, we demonstrated the nephroprotective effects of diosmin by targeting the mechanisms by which CYC-induced kidney damage occurs, as supported by a series of findings including the following: (i) the attenuation of CYC-induced histopathological changes; (ii) the reduction of pro-inflammatory cytokines; (iii) the mitigation of CYC-induced apoptosis, as reflected by the downregulation of BAX and the upregulation of Bcl-2 immunoreactivity; (iv) modulation of mTOR and SIRT1 by upregulation of mTOR and downregulation of SIRT1 immunoreactivity; and (v) improvement of cellular DNA damage.

Kidney sections from the CYC group exhibited marked histopathological alterations. In this study, CYC-induced renal histopathological changes, including necrosis and desquamation of epithelial cells, luminal dilatation of renal tubules, and enlargement of Bowman’s capsule, were consistent with previous reports (Abd El Salam et al. 2023; Çağlayan et al. 2018). In CYC, MT staining revealed increased collagen deposition (Tohamy et al. 2021), while PAS staining indicated reduced PAS positivity accompanied by disruption of the brush border. The damaging effects of CYC metabolites on cellular membranes are considered a hallmark of nephrotoxicity (Çağlayan et al. 2018) and are directly linked to its deleterious impact on renal function (Al-Attar et al. 2017). Consistent with these observations, our findings demonstrate that diosmin administration preserved renal histoarchitecture and attenuated CYC-induced renal injury. In a model of sodium arsenite-induced nephrotoxicity, diosmin at 100 mg/kg, as used in the present study, was reported to be the most effective dose for improving renal tissue damage (Mohtadi et al. 2023). The nephroprotective potential of diosmin has also been supported by studies in experimental models of renal injury induced by chemotherapeutic agents (Anwer et al. 2023; Abdel-Daim et al. 2017; Ali et al. 2021b), as well as metabolic disorders such as obesity (Zhu et al. 2024) and diabetes (Ahmed et al. 2016).

CYC-induced renal toxicity activates the NF-κB signaling pathway, which subsequently promotes the expression of pro-inflammatory cytokines (El-Kholy et al. 2017). ROS induce the dissociation of NFκB-p65 from IκB, resulting in nuclear localization and the subsequent induction of genes associated with inflammation, including TNF-α, IL-1β (Oztopuz et al. 2019), and iNOS (Ali et al. 2020). Our findings demonstrated a marked increase in the immunoreactivity intensities of pro-inflammatory markers, including IL-1β, IL-6, TNF-α, and iNOS, in the CYC-treated group. However, diosmin treatment significantly reduced the immunoreactivity of these inflammatory mediators.

The present results are consistent with previous studies reporting that diosmin attenuates inflammatory responses by reducing TNF-α, IL-1β, and IL-6 levels in experimental models of drug-induced toxicity (Abdel-Daim et al. 2017). Recent investigations have further highlighted the anti-inflammatory potential of diosmin, demonstrating its protective effects against gentamicin-induced nephrotoxicity (Geshnigani et al. 2023) and chlorpyrifos-induced neurotoxicity (Abd-Elhamid et al. 2024). Additionally, diosmin has been shown to confer renal protection by suppressing inflammatory pathways in models of chronic kidney injury, such as unilateral ureteral obstruction (Zhao et al. 2024).

CYC-induced nephrotoxicity is largely mediated by oxidative stress and inflammation, which promote cell death by activating caspase-3 and inhibiting Bcl-2 (Liu et al. 2016). The key proteins participating in the apoptotic mechanism, BAX and Bcl-2, are closely associated with the mitochondrial membrane (Gur et al. 2021). The function of the BAX in the apoptotic pathway is to enhance mitochondrial outer membrane permeability and facilitate the release of cytochrome C into the cytoplasm (Cosentino et al. 2022). Conversely, the Bcl-2 functions to prevent the process of cell death by inhibiting the release of cytochrome C and apoptosis-inducing factors (Basu 2022). Besides, the upregulation of NF-κB leads to an increase in BAX expression while concurrently reducing Bcl-2 expression (Zhang et al. 2017; Alaaeldin et al. 2020, 2021). The present findings demonstrate that diosmin normalized BAX and Bcl-2 immunoreactivity, supporting its anti-apoptotic potential in renal tissues. Consistent with our results, Perumal et al. (2018) reported that diosmin modulated apoptotic protein expression in an N-nitrosodiethylamine-induced hepatocellular carcinoma model.

One of the compounds responsible for CYC’s mutagenic activity, phosphoramide, has been demonstrated to induce cross-link formation and strand damage in DNA (Surh 2003; Hengstler et al. 1997). The present study investigated the DNA damage induced by the CYC’s active metabolites and evaluated the role of diosmin in mitigating this damage. The comet assay, a widely used technique for assessing DNA damage and repair, serves as a valuable tool in this context (Speit et al. 2009). Our findings indicate that CYC induced significant DNA damage, as evidenced by alterations in comet parameters. In contrast, diosmin treatment attenuated CYC-induced DNA damage. Consistent with our observations, previous comet assay analyses have demonstrated severe DNA damage following CYC exposure, characterized by increased tail length and elevated Tail DNA content (Tuorkey 2017).

A study using 8-OHdG, a marker of oxidative DNA damage, similarly reported a significant increase in the CYC group (Çağlayan et al. 2018). Another study demonstrated that diosmin pre-treatment attenuated radiation-induced DNA damage and reduced susceptibility to oxidative damage. This protective effect has been attributed to the antioxidant and antigenotoxic properties of diosmin (Mahgoub et al. 2020).

SIRT1, situated both within the cytoplasm and nucleus, is responsible for the deacetylation of specific histone and non-histone targets. As a multifunctional molecule, SIRT1 exerts cytoprotective effects against oxidative stress and various pathological conditions, including neurological, oncological, and renal diseases (Lohanathan et al. 2022). SIRT1 confers renoprotective effects primarily by modulating inflammatory, apoptotic, and fibrotic pathways, thereby attenuating the progression of renal injury (Morigi et al. 2018). In the present study, CYC treatment was associated with increased SIRT1 immunoreactivity, which may reflect a compensatory cellular response to oxidative stress. In contrast, combined CYC and diosmin treatment normalized SIRT1 immunoreactivity, yielding levels comparable to those observed in the control group. Increased SIRT1 expression has been reported in various pathological contexts, including sepsis-associated inflammation, where restoration of physiological SIRT1 balance was reported. Such observations suggest that elevated SIRT1 levels under conditions of cellular stress may represent compensatory or stress-adaptive responses rather than unequivocal anti-inflammatory activity. Accordingly, SIRT1 upregulation during tissue injury should not be interpreted as direct evidence of functional inflammation suppression (Smith et al. 2020). Hasegawa et al. (2010) demonstrated that experimental enhancement of SIRT1 expression exerted protective effects against cisplatin-induced acute kidney injury; however, no endogenous increase of SIRT1 was reported within the injury group, indicating that SIRT1-mediated protection was linked to controlled activation rather than injury driven upregulation. In contrast, Yalcin et al. (2023) observed elevated SIRT1 immunoreactivity in radiation-induced liver injury, supporting the view that SIRT1 expression may increase as a secondary response to tissue damage. Similarly, in ischemia/reperfusion-induced kidney injury, SIRT1 overexpression has been reported, where enhanced SIRT1 activity was associated with increased resistance to tissue damage. Conversely, the absence of functional SIRT1 aggravated renal injury, further underscoring the context-dependent role of SIRT1 under pathological conditions (Fan et al. 2013). Collectively, these findings reinforce the concept that SIRT1 alterations under pathological conditions may primarily reflect adaptive regulatory mechanisms associated with cellular stress and injury severity. This molecule exerts its regulatory function on cellular homeostasis via the activation of adenosine monophosphate-activated protein kinase and the inhibition of the mammalian target of rapamycin (mTOR) (Cetrullo et al. 2015). The mTOR signaling pathway can be initiated by the presence of circulating nutrients, growth factors, and pro-inflammatory cytokines (Buerger 2018; Marques-Ramos and Cervantes 2023). Reduced mTOR signaling is commonly associated with autophagy and may represent an adaptive response depending on cellular context. In the present model, however, mTOR suppression coincided with clear indicators of cellular stress and tissue injury, suggesting that this reduction may reflect stress-related signaling disturbances rather than a protective response.

Diosmin has been reported to attenuate inflammatory mediators, including IL-6, IL-1β, TNF-α, COX-2, and matrix metalloproteinases, in drug-induced toxicity models. Given that inflammatory signaling is a well-recognized upstream regulator capable of suppressing mTOR activity, the restoration of mTOR levels observed in the diosmin-treated groups may plausibly be attributed to a reduction in inflammatory burden (Mega et al. 2024). Consistent with this interpretation, diosmin has also demonstrated cytoprotective effects in gentamicin-induced nephrotoxicity, where it attenuated oxidative injury and preserved renal histoarchitecture (Ali et al. 2021b.). Collectively, these findings support the notion that the protective effects of diosmin may primarily involve mitigation of cellular stress, with mTOR normalization representing a secondary consequence of improved cellular homeostasis rather than direct pathway modulation. The conclusions of this study demonstrated that the mTOR immunoreactivity intensity was lowest in the CYC group, while treatment with diosmin significantly enhanced its immunoreactivity intensity. Experimental studies have also reported the downregulation of mTOR expression in CYC-induced kidney damage (Albayrak et al. 2018) and in different organs damaged by other anticancer drugs was demonstrated (Lee et al. 2015). Moreover, the aforementioned studies revealed elevated levels of mTOR expression in the antioxidant and damage + antioxidant groups (Abd El-Ghafar et al. 2021; Hassanein et al. 2020). Our findings align with previous research, proposing that the elevation in mTOR immunoreactivity observed following diosmin administration may stem from its protective effects against CYC-induced kidney damage.

SIRT1 may modulate mTOR activity, potentially through mechanisms involving the TSC1/TSC2 complex, a key upstream regulator of mTOR signaling (Ghosh et al. 2010). In addition, SIRT1 activation has been associated with modulation of the PI3K/mTOR signaling axis and with reductions in hypoxia-induced oxidative stress and inflammatory responses, as well as enhanced cardiomyocyte viability in experimental settings (Ma et al. 2022). In the present study, we evaluated the immunoreactivity and expression profiles of mTOR and SIRT1. Although our findings are consistent with previously reported functional associations, they do not establish a direct mechanistic interaction. Instead, the observed patterns likely represent coordinated regulation within broader stress-responsive signaling networks rather than a defined linear signaling cascade.

The present study has several limitations that should be acknowledged. The findings are derived from a controlled experimental animal model using a defined dosing protocol, which may not fully reflect the complexity of human disease. Species-specific differences in pharmacokinetics, metabolism, and pathophysiological responses may therefore limit direct generalizability to clinical settings. In addition, the evaluation of inflammatory, apoptotic, and signaling markers was primarily based on immunohistochemical analysis. Although this method enables spatial localization of protein expression within renal tissue, it provides semi-quantitative data and does not allow precise assessment of protein activation states or definitive conclusions regarding mechanistic causality. Consequently, while the results suggest favorable effects of diosmin, the underlying molecular mechanisms were not comprehensively characterized. Further quantitative, functional, and translational studies are needed to clarify mechanistic aspects and confirm clinical relevance.

## Conclusion

**Fig. 8:**
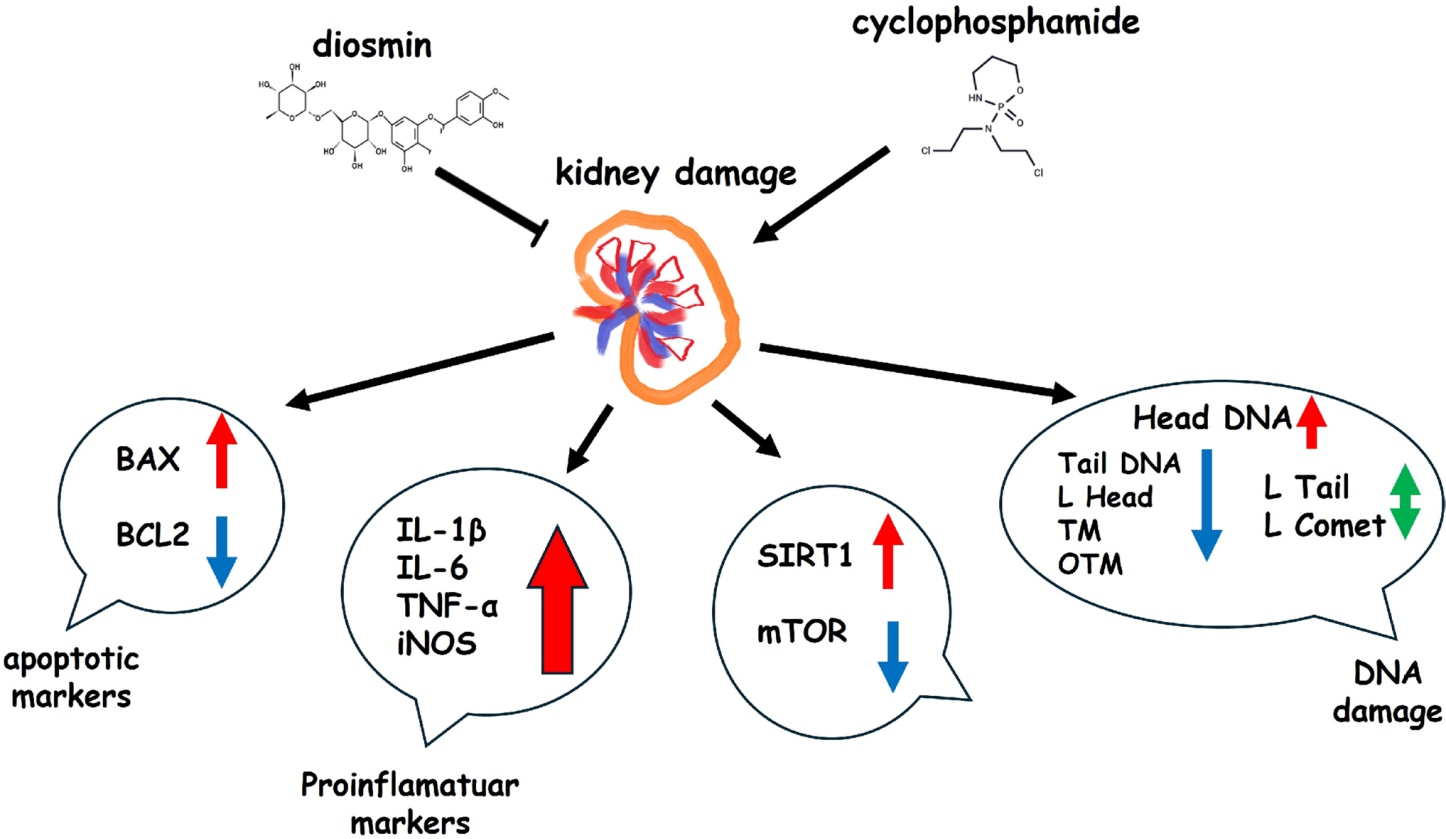
Mechanistic illustration of the effects of diosmin against CYC-induced kidney damage. Diosmin suppresses pro-inflammatory markers, promotes anti-apoptotic activity, attenuates cellular DNA damage, and modulates mTOR and SIRT1 immunoreactivity in the kidneys of CYC-treated rats

Inflammation, apoptosis, DNA damage, and alterations in mTOR and SIRT1 expression are closely associated with CYC-induced renal injury. In the present study, diosmin treatment was associated with reduced immunoreactivity of pro-inflammatory mediators (TNF-α, IL-1β, IL-6) and iNOS, suggesting attenuation of the inflammatory response. Diosmin was also associated with anti-apoptotic effects, as reflected by increased Bcl-2 and decreased BAX immunoreactivity. In addition, improvements in histological alterations and attenuation of DNA damage were observed. Taken together, these findings suggest that diosmin may exert favorable effects in CYC-induced renal injury within this experimental model. The observed modulation of inflammatory and apoptotic markers, along with improvements in histological features, supports the potential relevance of diosmin in attenuating CYC-associated renal alterations (Fig. 8).

## Data Availability

The data used to support the findings of this study are available from the corresponding author upon request.
